# A prospective study of clinical characteristics and outcomes of acute kidney injury in a tertiary care Centre

**DOI:** 10.1186/s12882-019-1466-z

**Published:** 2019-07-26

**Authors:** Su Hooi Teo, Kian-Guan Lee, Riece Koniman, Alvin Ren Kwang Tng, Zhong Hong Liew, Thin Thiri Naing, Huihua Li, Ru Yu Tan, Han Khim Tan, Hui Lin Choong, W. Y. Marjorie Foo, Manish Kaushik

**Affiliations:** 1 Department of Renal Medicine, Academia, Singapore General Hospital, 20 College Road, Singapore, 169856 Singapore; 2 Health Services Research Unit, Bachelor of Nursing, University of Sydney, Singapore General Hospital, Singapore, Singapore; 3 Centre for Quantitative Medicine, Duke-NUS Medical School, Singapore, Singapore

**Keywords:** Acute kidney injury, Dialysis, Mortality

## Abstract

**Background:**

Acute kidney injury (AKI) is a major global health problem. We aim to evaluate the epidemiology, risk factors and outcomes of AKI episodes in our single centre.

**Methodology:**

We prospectively identified 422 AKI and acute on chronic kidney disease episodes in 404 patients meeting KDIGO definitions using electronic medical records and clinical data from 15th July to 22nd October 2016, excluding patients with baseline estimated GFR (eGFR) of < 15 mL/min. Patients were followed up till 6 months after AKI diagnosis.

**Results:**

The mean age was 65.8 ± 14.1. Majority of patients were male (58.2%) of Chinese ethnicity (68.8%). One hundred and thirty-two patients (32.6%) were diagnosed in acute care units. Seventy-five percent of patients developed AKI during admission in a non-Renal specialty. Mean baseline eGFR was 50.2 ± 27.7 mL/min. Mean creatinine at AKI diagnosis was 297 ± 161 μmol/L. Renal consultations were initiated at KDIGO Stages 1, 2 and 3 in 58.9, 24.5 and 16.6% of patients, respectively. Three hundred and ten (76.7%) patients had a single etiology of AKI with the 3 most common etiologies of AKI being pre-renal (27.7%), sepsis-associated (25.5%) and ischemic acute tubular necrosis (15.3%). One hundred and nine (27%) patients received acute renal replacement therapy. In-hospital mortality was 20.3%. Six-month mortality post-AKI event was 9.4%. On survival analysis, patients with KDIGO Stage 3 AKI had significantly shorter survival than other stages.

**Conclusion:**

AKI is associated with significant in-hospital to 6-month mortality. This signifies the pressing need for AKI prevention, early detection and intervention in mitigating reversible risk factors in order to optimize clinical outcomes.

**Electronic supplementary material:**

The online version of this article (10.1186/s12882-019-1466-z) contains supplementary material, which is available to authorized users.

## Background

Acute kidney injury is one of the major complications in acutely ill patients and imposes significant mortality and morbidity globally [1–5]. AKI may be present on admission to hospital or develop during the course of hospitalization [6]. Based on the Kidney Disease, Improving Global Outcomes (KDIGO) report, the incidence of AKI in hospitalized patients ranges from 17 to 31% [7–9]. AKI-related inpatient care is also associated with increased healthcare costs due to prolonged hospitalizations, additional investigations and the development of complications such as the need for renal replacement therapy (RRT), cardiovascular complications and re-admissions [10–12]. The 2009 National Confidential Enquiry into Patient Outcomes and Death (NCEPOD) reported that 50% of patients who died from AKI received suboptimal care and 14% of AKI was avoidable [13]. In 2013, the International Society of Nephrology launched the 0by25 initiative of improving timely diagnosis and treatment of AKI globally with an aim to eliminate preventable deaths from AKI worldwide by 2025 [8]. In view of this, a considerably greater attention has been paid to Asian countries. The risk factors, myriad of etiologies and consequences of AKI have been well-delineated [6, 14, 15]. Given the diversity in culture, ethnicity, climate and socioeconomic status, it is not surprising that a difference in etiology, incidence and risk factors of AKI exists in various parts of Asia. The pooled- incidences of AKI in hospitalized patients in Asia vary from 9.0% in Central Asia to 31.0% in Southeastern Asia [7]. The development of AKI has been shown to progress to chronic kidney disease (CKD) and end stage renal disease (ESRD). To date, the data on acute kidney injury in Singapore is scattered. Therefore, we aimed to (i) analyze the distribution of AKI in different clinical units of adults admitted to a hospital in Singapore, and (ii) describe the clinical characteristics, risk profiles and outcomes of AKI.

## Methods

### Study population

Data on patients referred to the Nephrology Department in Singapore General Hospital (a 1785-bedded tertiary hospital) and diagnosed with AKI by KDIGO (2012) criteria from 15th July to 22nd October 2016 were prospectively collected from electronic medical records and clinical notes. Patients with estimated glomerular filtration rate (eGFR) of ≤15 mL/min were excluded. The study protocol was approved by the SingHealth Centralized Institutional Review Board (IRB). Patients were followed up till 6 months after AKI diagnosis.

### Definition

We studied AKI according to the KDIGO 2012 AKI criteria, (i) increase in serum creatinine ≥26.5 μmol/L within 48 h, (ii) increase in serum creatinine ≥1.5x from baseline serum creatinine within the prior 7 days. Baseline serum creatinine was defined as the result on admission or the latest available serum creatinine within the preceding 12 months prior to admission, whichever available. Patients were included if serum creatinine fulfilled criteria for minimum KDIGO 2012 stage within 24 h of admission. Hypotension preceding diagnosis of AKI was defined as mean arterial pressure (MAP) of less than 70 mmHg or the use of inotropes or vasopressors.

### Statistical analysis

Mean and standard deviation (SD) were reported for continuous variables, while frequency and proportion were reported for categorical data. Overall survival was defined from the date of AKI diagnosis to the date of death, or last follow-up date for censored cases. Overall survival was estimated by the Kaplan–Meier method. Log-rank test was used to compare survival curves. Univariable Cox regression was carried out to evaluate the effects of potential factors on overall survival. All the variables with *p*-value of < 0.2 by univariable Cox regression were included in the multivariable analysis. Multivariable model was built up by means of reduced model selection using Akaike’s information criterion (AIC). For all analyses, *p* value is taken as statistically significant when it is < 0.05. R 3.4.2 (https://www.r-project.org) was used for analysis.

## Results

### Clinical characteristics of AKI patients

A total of 422 episodes of AKI in 404 patients were identified to have AKI. The clinical characteristics of the patients are shown in Table 1. The severity of AKI was classified as KDIGO AKI Stage 1 in 238 (58.9%) patients; Stage 2 in 99 (24.5%) patients and Stage 3 in 67 (16.6%) patients. Among critically ill patients, 89 (22%) patients with AKI were detected whilst in Intensive Care Unit (ICU) and 43 (10.6%) in Intermediate Care Area (ICA) or High Dependency Units. Two hundred seventy-two (67.3%) patients developed AKI in the general wards, of which 67% were in Stage 1. In our study, 147 (36.1%) patients with AKI were detected in the medical departments, while 81 (20%) were detected in the cardiac units and 78 (19.3%) in the surgical departments. Ninety-eight (24.3%) patients with AKI were identified in the nephrology unit.

**Table 1:** Baseline Characteristics of the study cohort (per patient, total no = 404)

Characteristics	Mean (SD)
Age	65.8 (14.1)
BMI	24.7 (5.3)
Baseline creatinine	150 μmol/L (71)
Baseline eGFR	50.2 mL/min (27.7)
Creatinine at AKI diagnosis	297.5 μmol/L (160.7)
Gender (%)	
Male	235 (58.2)
Female	169 (41.8)
Ethnicity (%)	
Chinese	278 (68.8)
Malay	71 (17.6)
Indian	39 (9.7)
Others	16 (4)
Primary Etiology of AKI	
Pre-renal	112 (27.7)
Ischemic acute tubular necrosis	62 (15.3)
Cardio-renal syndrome	33 (8.2)
Hypertensive emergency	18 (4.5)
Allograft rejection	1 (0.2)
Abdominal compartment syndrome	2 (0.5)
Tubular obstruction	9 (2.2)
Drug- associated AKI	12 (3)
Cardiac surgery associated-AKI	20 (5)
Sepsis associated-AKI	105 (26)
Contrast-induced AKI (CIN)	7 (1.7)
Obstructive uropathy	9 (2.2)
Glomerulonephritis	10 (2.5)
Hepatorenal syndrome	4 (1)
Single or multiple etiology	
Single	310 (76.7)
Multiple	94 (23.3)
Location	
General wards	272 (67.3)
Intensive care area/ High dependency unit	43 (10.6)
Intensive care unit	89 (22)
Characteristics	Frequency (%)
Service where patient is located	
Cardiac	81 (20)
Surgery	78 (19.3)
Medical	147 (36.1)
Renal	98 (24.3)
Comorbidities	
Diabetes Mellitus	230 (56.9)
Hypertension	302 (74.8)
Cardiovascular disease	187 (46.3)
Atrial fibrillation	58 (14.4)
Cerebrovascular accident	57 (14.1)
Cancer	78 (19.3)
KDIGO stage at AKI diagnosis	
1	238 (58.9)
2	99 (24.5)
3	67 (16.6)

Of the 404 patients, 235 (58.2%) were male and 169 (41.8%) were female. The mean age was 65.8 ± 14.1 years, with 58.9% older than 65 years. The ethnicity distribution reflected that of our hospital population as a whole. Mean baseline serum creatinine was 150 ± 71 μmol/L, with a corresponding eGFR of 50 ± 27.7 mL/min. With regards to comorbidities, 302 (74.8%) had hypertension, 230 (56.9%) had diabetes mellitus, and 187 (46.3%) had ischemic heart disease. Seventy-eight cases (19.3%) of AKI occurred in the background of underlying malignancy. Mean serum creatinine at AKI diagnosis was 297.5 ± 160.7 μmol/L. The most frequent cause of AKI was noted to be pre-renal cause, with an occurrence in 112 (27.7%) patients, followed by sepsis-associated AKI and ischemic acute tubular necrosis (ATN) occurring in 105 (26%) and 62 (15.3%) patients, respectively. Dialysis was carried out in 109 (27%) of our AKI patients, of which 62 (56.9%) received continuous renal replacement therapy (CRRT).

### Univariable analysis

The univariate analysis of risk factors associated with mortality for patients with AKI is shown in Table 2. Elderly patients had a statistically significant shorter survival (HR 1.54, 95% 1.07–2.22, *p* = 0.0201). Malay ethnicity was noted to have a lower risk of mortality (HR 0.55, 95% CI 0.33–0.94, *p* = 0.0272). Baseline eGFR of > 60 mL/min was associated with a higher risk of mortality (HR 1.54, 95% CI 1.08–2.21, *p* = 0.0180). Renal transplant recipients had a lower risk of death (HR 0.11, 95% CI 0.03–0.46, *p* = 0.0023). Paradoxically, hypertension was found to be associated with a lower mortality in AKI (HR 0.5, 95% CI 0.35–0.72, *p* = 0.0002). Multifactorial AKI was also found to be associated with higher mortality (HR 1.81, 95% CI 1.25–2.61, *p* = 0.0017). Patients with hypernatremia had a lower survival (HR 2.31, 95% CI 1.21–4.38, p = 0.0180). Presence of hypotension in the preceding 48 h prior to occurrence of AKI was strongly associated with mortality (HR 3.35, 95% 2.18–5.13, *p* < 0.0001). There was almost a twofold increased risk of mortality in AKI Stage 3 (HR 1.88, 95% CI 1.23–2.88, *p* = 0.0034). AKI patients who required renal replacement therapy had a significantly higher mortality (HR 2.74, 95% CI 1.93–3.89, *p* < 0.001).

**Table 2:** Univariate Analysis of Risk Factors associated with Mortality for Patients with AKI

	HR (95% CI)	*P* value
Age		
≤ 65	Reference	
> 65	1.54 (1.07, 2.22)	0.0201
BMI		
≤ 30	Reference	
> 30	0.97 (0.57, 1.65)	0.9138
Baseline eGFR (mL/min)		
≤ 60	Reference	
> 60	1.54 (1.08, 2.21)	0.0180
Urea at RRT Initiation (mmol/L)		
≤ 30	Reference	
> 30	0.82 (0.48, 1.39)	0.4533
Sodium at RRT Initiation (mmol/L)		
≤ 146	Reference	
> 146	2.31 (1.21, 4.38)	0.0108
Serum potassium at RRT Initiation (mmol/L)		
≤ 5	Reference	
> 5	0.9 (0.51, 1.58)	0.7028
Serum chloride at RRT Initiation (mmol/L)		
≤ 107	Reference	
> 107	1.02 (0.59, 1.77)	0.9327
Serum bicarbonate at RRT Initiation (mmol/L)		
≤ 19	Reference	
> 19	1.15 (0.69, 1.94)	0.5847
Serum albumin at RRT Initiation (g/dL)		
≤ 40	Reference	
> 40	5.03 (0.67, 37.6)	0.1153
Hemoglobin at RRT Initiation (g/dL)		
≤ 10	Reference	
> 10	0.74 (0.43, 1.28)	0.2841
Serum lactate at RRT Initiation (mmol/L)		
≤ 2.2	Reference	
> 2.2	1.75 (0.98, 3.13)	0.0601
Gender		
Male	Reference	
Female	1.12 (0.79, 1.58)	0.5370
Ethnicity		
Chinese	Reference	
Malay	0.55 (0.33, 0.94)	0.0272
Indian	0.52 (0.25, 1.07)	0.0775
Others	0.71 (0.26, 1.94)	0.5059
	HR (95% CI)	P value
Renal transplant		
No	Reference	
Yes	0.11 (0.03, 0.46)	0.0023
Diabetes mellitus		
No	Reference	
Yes	0.83 (0.59, 1.18)	0.3062
Hypertension		
No	Reference	
Yes	0.5 (0.35, 0.72)	0.0002
Cardiovascular disease		
No	Reference	
Yes	1.22 (0.87, 1.73)	0.2538
Atrial fibrillation		
No	Reference	
Yes	1.4 (0.89, 2.2)	0.1444
Cerebrovascular accident		
No	Reference	
Yes	0.95 (0.58, 1.57)	0.8555
Cancer		
No	Reference	
Yes	1.29 (0.85, 1.93)	0.2294
Any hypotension in the preceding 48 h		
No	Reference	
Yes	3.35 (2.18, 5.13)	< 0.0001
NA	1.1 (0.67, 1.8)	0.7208
KDIGO stage at AKI diagnosis		
1	Reference	
2	1.04 (0.67, 1.61)	0.8646
3	1.88 (1.23, 2.88)	0.0034
Single or multiple etiology of AKI		
Single	Reference	
Multiple	1.81 (1.25, 2.61)	0.0017
Did the patient receive RRT during admission		
No	Reference	
Yes	2.74 (1.93, 3.89)	< 0.0001

### Multivariable analysis

In the multivariable analysis shown in Table 3, the independent baseline variable that was significantly associated with mortality was age more than 65 (HR 1.46, 95% CI 1.00–2.13, *p* = 0.0483). Interestingly, AKI in the setting of renal transplant recipients (HR 0.17, 95% CI 0.04–0.70, *p* = 0.0143) and hypertension (HR 0.53, 95% CI 0.37–0.78, *p* = 0.0010) were significantly associated with a lower risk of mortality. Presence of hypotension in the preceding 48 h (HR 2.57, 95% CI 1.63–4.07, *p* = 0.0001) prior to development of AKI and AKI requiring dialysis (HR 1.67, 95% CI 1.14–2.44, *p* = 0.0084) were significantly associated with death.

**Table 3:** Multivariate Analysis of Risk Factors associated with Mortality for Patients with AKI

	HR (95% CI)	P value
Age		
≤ 65	Reference	
> 65	1.46 (1.00, 2.13)	0.0483
Renal Transplant		
No	Reference	
Yes	0.17 (0.04, 0.70)	0.0143
Hypertension		
No	Reference	
Yes	0.53 (0.37, 0.78)	0.0010
Any hypotension in the preceding 48 h		
No	Reference	
Yes	2.57 (1.63, 4.07)	0.0001
NA	1.47 (0.88, 2.44)	0.1375
Did the patient receive RRT during admission		
No	Reference	
Yes	1.67 (1.14, 2.44)	0.0084

Risk factors associated with mortality based on one episode of AKI per patient are presented in the Additional file 1: Table S1 and Table S2.

### Outcomes of AKI

The outcomes associated with AKI are shown in Table 4. Median serum creatinine at discharge was 176 μmol/L (IQR 114-278 μmol/L). Upon discharge, 16 (4%) patients still required dialysis support. In-hospital mortality rate was 20.3% while 6-month mortality was 9.4%.

**Table 4:** Clinical outcomes of patients with AKI

Outcomes	Patients (*n* = 404) (%)
Total in-hospital mortality	82 (20.3)
Received RRT during admission	109 (27)
Required dialysis support upon discharge	16 (4)
Median serum creatinine on discharge (μmol/L)	176 (IQR 114-278 μmol/L)
Initial modality	
Intermittent hemodialysis	6 (1.5)
Sustained low efficiency dialysis (SLED)	41 (10.1)
Continuous renal replacement therapy (CRRT)	62 (15.3)
6-month mortality	38 (9.4)

The survival rates at 3- and 6-months post-AKI diagnosis according to severity of AKI, are shown in Table 5. AKI was associated with decreased survival according to severity of AKI stages at 3 months post-AKI episode (75.9, 95% CI 70.7–81.5% for Stage 1; 73.7, 95% CI 65.3–83.1% for Stage 2, and 57.4, 95% CI 46.7–70.4% for Stage 3). There were significant differences in survival between the severity of AKI stages (log-rank test *p*-value 0.0091).

**Table 5:** Survival rate 3 and 6 months post-AKI diagnosis according to severity of AKI

	Survival rate (95% CI)	
KDIGO stage	3 months survival	6 months survival
1	75.9% (70.7, 81.5%)	71.4% (65.9, 77.3%)
2	73.7% (65.3, 83.1%)	70.5% (61.9, 80.3%)
3	57.4% (46.7, 70.4%)	54.4% (43.8, 67.6%)

**Fig. 1:**
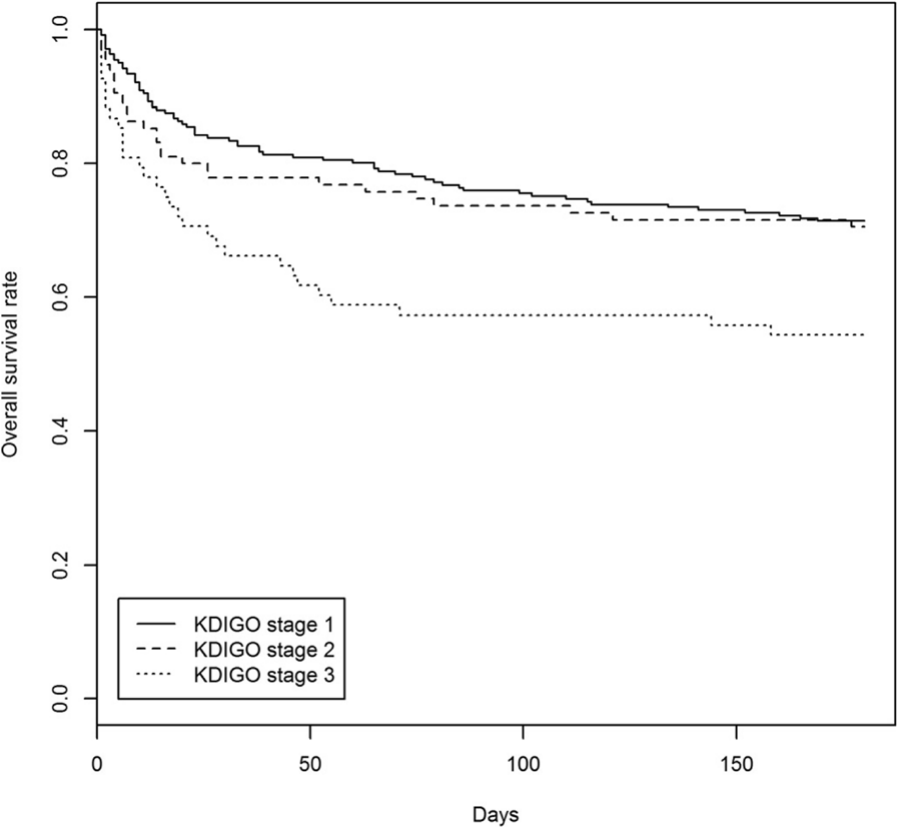
Kaplan-Meier estimated survival for severity of AKI. Log-rank test p-value = 0.0091

At 6 months, AKI was associated with decreased survival with worsening severity of AKI stages: 71.4% (95% CI 65.9–77.3%) for Stage 1, 70.5% (95% CI 61.9–80.3%) for Stage 2, 54.4% (95% CI 43.8–67.6%) for Stage 3 (log-rank test p-value 0.0091). Fig. 1 shows the Kaplan-Meier estimated survival according to AKI stages. On survival analysis, patients with KDIGO Stage 1 and 2 AKI had significantly better survival than Stage 3 AKI.

## Discussion

This study investigated a cohort of 404 patients admitted to a tertiary hospital over 100 days. Patients with AKI were predominantly identified in non-nephrology specialties, and nephrologists’ involvement started after consultation was requested. Sixty percent (60%) of referrals for AKI were at Stage 1 AKI at point of nephrology consult. We have demonstrated that AKI developed in 22% of critically ill patients. This is consistent with other studies showing incidence rate of AKI during ICU stays varying from 22 to 67% [16–19]. The epidemiological data, clinical features and etiology on AKI in Asian countries differ from that of what we found in our data, especially with regards to precipitants of AKI. To-date, there is a paucity of data from Singapore on clinical characteristics, etiologies and outcomes of patients with AKI. Our study was intended to give us an insight into this. Locally, Chua et al. evaluated 207 patients with septic AKI and mortality in Singapore and reported that a 1-year mortality rate of 40%, with high daily fluid balance and frusemide administration being modifiable risk factors [20]. In terms of cardiac surgery-associated AKI in South-East Asian population, Chew et al. reported that 35.3% of patients developed AKI after cardiac surgery, with Indian and Malay ethnicity having a higher risk than Chinese ethnicity [21]. Our study provides novel facts on the major affected clinical settings of AKI, clinical characteristics, risk factors and outcomes associated with AKI across different etiological insults.

We identified pre-renal cause as a precipitant of AKI in 27.7%, a lower proportion compared to a study by Tang et al. reporting pre-renal cause of AKI of 49.1% from the medical departments [22]. Volume resuscitation to replace ongoing losses and restoration to baseline volume status is crucial in the event of true extracellular fluid volume depletion. Knowledge of baseline weight, careful attention to intake and output and ongoing serial weight measurement and volume status assessment directs the strategy for resuscitation [23, 24].

In our centre, 27% of AKI patients received acute renal replacement therapy (RRT), of which 56.9% received CRRT. Our finding was relatively consistent with a study by Yang et al. reporting that 22.5% of patients received RRT [25]. The initiation of RRT in patients with severe AKI contributes to healthcare costs and is a measure of complexity of care in critically ill patients. The practice of prescription of CRRT therapy varies widely across different continents. In a retrospective analysis of 261 patients in 2 ICUs in Canada estimating the cost comparison between CRRT and intermittent hemodialysis, the weekly cost ranged from 3,486 to 5,117 Canadian dollars (depending on modality choice and anticoagulation) [26]. This cost was significantly more expensive than intermittent hemodialysis at a weekly cost of 1,342 Canadian dollars. The frequency of dialysis requirement in AKI ranges from 6.9% in Asia to 71% in the study by Beginning and Ending Supportive Therapy for the Kidney (BEST Kidney) Investigators [19] [27],

Acute kidney injury remains a common clinical problem, particularly in the elderly. Liano et al. reported a mean age of 64 years in their study involving AKI episodes occurring in adult patients admitted to any of the 13 tertiary care hospitals in Madrid [28]. The mean age of patients was 73 in two UK district hospitals reported by Meran et al [29] and 64.7 in a separate Canadian-based ICU study [30]. Similarly, patients in our study had a median age of 65.8 years. We demonstrated that patients with AKI who were 65 years old and above were associated with higher risks of mortality. Age older than 65 is not only a risk factor for impaired recovery from AKI and progression to advanced-stage CKD, but the long-term survival of patients with AKI worsens with increasing age, even in non-dialysis requiring AKI [31–33]. In the setting of reduced renal reserve in the elderly [34], the higher frequency of nephrotoxins usage such as non-steroidal anti-inflammatory drugs predisposes these patients to AKI [34]. The incidence of AKI in the elderly population is expected to be on the rise given the challenges of the elderly population in the nation, along with an interplay of polypharmacy and susceptibility to nephrotoxic agents in the aged population.

In previously reported studies on transplant AKI epidemiology, the diagnosis of AKI was based on RIFLE criteria [35]. In our study, we analyzed the incidence of AKI meeting the definition in KDIGO 2012. Mehrotra et al. included 27, 232 kidney transplant recipients of which 11.3% developed AKI during the study period and it was reported that patients who developed AKI had an increased risk of death (HR 2.36, 92% CI 2.41–2.60) [36]. However, hazard ratios for both outcomes of graft loss and death were inversely related to the severity of CKD. In our study, AKI in renal transplant recipients was associated with a lower risk of mortality paradoxically. The subgroup of the renal transplant recipients in our study was younger and had a baseline eGFR > 30 mL/min. Sepsis-associated AKI (SA-AKI) was the leading precipitant of AKI in renal transplant recipients, with the majority not requiring dialysis. Care of renal transplant recipients in our institution is provided by a dedicated renal transplant team, and any SA-AKI was aggressively managed with input from transplant infectious disease specialists. These factors may potentially contribute to a significantly lower risk of mortality in this group of AKI patients but have yet to be validated.

The identification of risk factors predicting risk of mortality is imperative so that early mitigating factors can be implemented. Many studies have attempted to identify prognostic factors in predicting AKI in critically ill patients [37, 38]. Previous reports have demonstrated that oliguria, pre-existing CKD, AKI attributable to nephrotoxic agents, AKI severity, and multi-organ failure were significant independent risk factors for death in AKI patients. In the Madrid Acute Renal Failure Study Group by Liano et al., oliguria, sustained hypotension, assisted respiration and icterus were associated with higher mortality [28]. Interestingly in our study, an underlying comorbidity of hypertension was not significantly associated with mortality. This finding may potentially be related to the counter-effect of hypertension against ischemic insult during an episode of AKI and it remains to be validated in future studies.

Hemodynamic instability is one of the most common causes of acute kidney injury. Our understanding of kidneys receiving about 25% of our cardiac output, allows us to target adequate renal perfusion as a potential strategy to modify the risk of developing AKI. Although the mean arterial pressure (MAP) target of ≥65 mmHg was defined in the Surviving Sepsis Campaign Guideline 2018, a recent study by Saito and colleagues had measured hemodynamic pressure-related parameters comparing between patients with progression of AKI versus those without AKI progression [39]. The authors measured hemodynamic pressure-related parameters including systolic arterial pressure (SAP), diastolic arterial pressure (DAP), MAP and central venous pressure (CVP), mean perfusion pressure (MPP) and diastolic perfusion pressure (DPP) and calculated deficits in the above values. The study observed a significant difference in the DPP, MPP and DAP in the patients with AKI progression, and suggested that these deficits may potentially be modifiable risk factors for the prevention of AKI progression, particularly in the patients who had undergone cardiac surgery. In our study, we found that AKI patients with hypotension in the preceding 48 h prior to development of AKI were reported to be strongly associated with mortality. Silva et al. reported a similar finding of hypotension being an independent risk factor for death in the intensive care units [40].

The risk of mortality with severe AKI requiring RRT remains high, particularly in the setting of critical illness, estimated to be approaching 60% [41, 42]. Factors such as AKI stage, severity of acute non-renal organ dysfunction and underlying diagnosis were associated with increased risk for mortality after AKI [43]. Our study showed that 109 patients (27%) required RRT during admission. Hsu et al. also demonstrated that the incidence of dialysis-requiring AKI had been escalating rapidly, averaging at 10% annually in the United States with similar trends observed worldwide [44, 45]. An episode of dialysis-requiring AKI was a strong independent risk factor for long-term risk of progressive CKD and mortality [46]. Our finding of AKI-requiring dialysis as an independent predictor for mortality is consistent with previous literature [46–48].

The major strengths of our study are the detailed evaluation of the risk factors and the distribution of AKI within clinical departments. However, our findings should be interpreted in light of the following limitations. The definition of AKI used in our study was based on serum creatinine change unaccompanied by urinary output, hence leading to underestimation of the detection rate of AKI. Secondly, the study may run an inherent risk of sampling bias as it was conducted over a snapshot period. Thirdly, as the baseline serum creatinine method has not been well-unified, method such as using the minimum value of preadmission serum creatinine as a baseline creatinine has been shown to identify more patients with AKI and yield better predictive ability for 60-day mortality [49]. Multiple AKI biomarkers that are measured in the urine or plasma of patients with AKI have been discovered, including the neutrophil gelatinase-associated lipocalin (NGAL), kidney injury molecule 1 (KIM-1), liver-type fatty acid-binding protein (L-FABP), interleukin 18 (IL-18), calprotectin, urine angiotensinogen (AGT), urine microRNAs and the recently FDA-approved insulin-like growth factor-binding protein 7 x tissue inhibitor of metalloproteinase 2 in the USA [50]. Biomarkers for AKI diagnosis are not currently being used routinely in our local clinical practice, hence our study did not include any novel biomarkers for AKI diagnosis. In our future research, we hope to leverage the relationship of biomarkers in diagnosing AKI and predicting short and long-term outcomes of acute kidney injury in different patient care settings, given the heterogeneity of this condition. Finally, this study did not include the long-term outcomes of patient survival and the risk of ESRD after 6 months.

## Conclusion

In conclusion, our study shows that AKI resulted in an in-hospital mortality of 20.3%. Additionally, the AKI survivors had a mortality risk of 9.4% at 6 months. Risk factors including age above 65, presence of hypotension in the preceding 48 h prior to the development of AKI and AKI requiring dialysis were significantly associated with mortality. The data we have presented will enable policies to be drawn and healthcare costs to be quantified. Thus, these findings highlight the urgent need to develop effective treatments, explore educational opportunities pertaining to AKI, and improve hospital-based care processes aimed at early identification to prevent devastating outcomes.

## Additional file

Additional file 1: **Table S1.** Univariable Analysis of Risk Factors associated with Mortality for Patients with AKI (based on one episode of AKI per patient). **Table S2.** Multivariable Analysis of Risk Factors associated with Mortality for Patients with AKI (based on one episode of AKI per patient). (DOCX 22 kb)

## Data Availability

All datasets generated and/or analysed during the current study are not publicly available due to confidentiality of the data but are available from the corresponding author on reasonable request.

## Abbreviations

AIC: Akaike’s information criterion
AKI: Acute Kidney Injury
ATN: Acute tubular necrosis
CI: Confidence interval
CKD: Chronic kidney disease
CRRT: Continuous renal replacement therapy
DAP: Diastolic arterial pressure
DPP: Diastolic perfusion pressure
eGFR: Estimated glomerular filtration rate
ESRD: End stage renal disease
HR: Hazard ratio
ICA: Intermediate Care Area
ICU: Intensive Care Unit
IRB: Institutional Review Board
KDIGO: Kidney Disease, Improving Global Outcomes
MAP: Mean arterial pressure
MPP: Mean perfusion pressure
NCEPOD: National Confidential Enquiry into Patient Outcomes and Death
RIFLE: Risk Injury, Failure, Loss of kidney function, End-stage kidney disease
RRT: Renal replacement therapy
SA-AKI: Sepsis-associated acute kidney injury
SAP: Systolic arterial pressure
SD: Standard deviation
