# Supplementary material for: A prospective study of clinical characteristics and outcomes of acute kidney injury in a tertiary care Centre
Source: BMC Nephrol. 2019 Jul 26;20:282. doi: 10.1186/s12882-019-1466-z (PMC6660929; doi:10.1186/s12882-019-1466-z)
Supplement: Supplementary file 1 — Table S1. Univariable Analysis of Risk Factors associated with Mortality for Patients with AKI (based on one episode of AKI per patient). Table S2. Multivariable Analysis of Risk Factors associated with Mortality for Patients with AKI (based on one episode of AKI per patient). (DOCX 22 kb) [file 12882_2019_1466_MOESM1_ESM.docx]

Table 6: Univariable Analysis of Risk Factors associated with Mortality for Patients with AKI (based on one episode of AKI per patient)

|  | HR (95% CI) | P value |
| --- | --- | --- |
| Age |  |  |
| ≤65 | Reference |  |
| >65 | 1.54 (1.07, 2.22) | **0.0197** |
| BMI |  |  |
| ≤30 | Reference |  |
| >30 | 0.97 (0.57, 1.65) | 0.9190 |
| Baseline eGFR (mL/min) |  |  |
| ≤60 | Reference |  |
| >60 | 1.55 (1.08, 2.22) | **0.0168** |
| Urea at RRT Initiation (mmol/L) |  |  |
| ≤30 | Reference |  |
| >30 | 0.79 (0.46, 1.35) | 0.3880 |
| Sodium at RRT Initiation (mmol/L) |  |  |
| ≤146 | Reference |  |
| >146 | 2.13 (1.1, 4.15) | **0.0257** |
| Serum potassium at RRT Initiation (mmol/L) |  |  |
| ≤5 | Reference |  |
| >5 | 0.87 (0.49, 1.56) | 0.6470 |
| Serum chloride at RRT Initiation (mmol/L) |  |  |
| ≤107 | Reference |  |
| >107 | 0.91 (0.51, 1.60) | 0.7360 |
| Serum bicarbonate at RRT Initiation (mmol/L) |  |  |
| ≤19 | Reference |  |
| >19 | 1.06 (0.63, 1.8) | 0.8250 |
| Serum albumin at RRT Initiation (g/dL) |  |  |
| ≤40 | Reference |  |
| >40 | 5.36 (0.72, 40.10) | 0.1020 |
| Hemoglobin at RRT Initiation (g/dL) |  |  |
| ≤10 | Reference |  |
| >10 | 0.73 (0.42, 1.27) | 0.2640 |
| Serum lactate at RRT Initiation (mmol/L) |  |  |
| ≤2.2 | Reference |  |
| >2.2 | 1.64 (0.91, 2.97) | **0.0979** |
| Gender |  |  |
| Male | Reference |  |
| Female | 1.12 (0.79, 1.58) | 0.5370 |
| Ethnicity |  |  |
| Chinese | Reference |  |
| Malay | 0.55 (0.32, 0.93) | **0.0265** |
| Indian | 0.52 (0.25, 1.07) | 0.0764 |
| Others | 0.71 (0.26, 1.94) | 0.5098 |
|  |  |  |
|  | HR (95% CI) | P value |
| Renal transplant |  |  |
| No | Reference |  |
| Yes | 0.11 (0.03, 0.46) | **0.0022** |
| Diabetes mellitus |  |  |
| No | Reference |  |
| Yes | 0.82 (0.58, 1.17) | 0.2750 |
| Hypertension |  |  |
| No | Reference |  |
| Yes | 0.5 (0.35, 0.72) | **0.0002** |
| Cerebrovascular accident |  |  |
| No | Reference |  |
| Yes | 1.27 (0.89, 1.79) | 0.1840 |
| Atrial fibrillation |  |  |
| No | Reference |  |
| Yes | 1.39 (0.88, 2.18) | 0.1550 |
| CVA |  |  |
| No | Reference |  |
| Yes | 0.95 (0.58, 1.57) | 0.8510 |
| Cancer |  |  |
| No | Reference |  |
| Yes | 1.29 (0.86, 1.95) | 0.2160 |
| Any hypotension in the preceding 48 hours |  |  |
| No | Reference |  |
| Yes | 3.41 (2.22, 5.22) | **<0.0001** |
| NA | 1.12 (0.68, 1.85) | 0.6510 |
| KDIGO stage |  |  |
| 1 | Reference |  |
| 2 | 1.01 (0.65, 1.57) | 0.9588 |
| 3 | 1.91 (1.25, 2.91) | **0.0029** |
| Single or multiple etiology |  |  |
| Single | Reference |  |
| Multiple | 1.81 (1.25, 2.62) | **0.0016** |
| Did the patient receive RRT during admission |  |  |
| No | Reference |  |
| Yes | 2.83 (1.99, 4.01) | **<0.0001** |

Table 7: Multivariable Analysis of Risk Factors associated with Mortality for Patients with AKI (based on one episode of AKI per patient)

|  | HR (95% CI) | P value | |
| --- | --- | --- | --- |
| Ethnicity |  | |  |
| Chinese | Reference | |  |
| Malay | 0.55 (0.32, 0.94) | | **0.0276** |
| Indian | 0.59 (0.28, 1.17) | | 0.1269 |
| Others | 0.62(0.22, 1.72) | | 0.3609 |
| Renal transplant |  | |  |
| No | Reference | |  |
| Yes | 0.14 (0.03 0.57) | | **0.0064** |
| Hypertension |  | |  |
| No | Reference | |  |
| Yes | 0.58 (0.40, 0.84) | | **0.0037** |
| Any hypotension in the preceding 48 hours |  | |  |
| No | Reference | |  |
| Yes | 2.52 (1.60, 3.98) | | **0.0001** |
| NA | 1.53 (0.92, 2.54) | | 0.1027 |
| Did the patient receive RRT during admission |  | |  |
| No | Reference | |  |
| Yes | 1.73 (1.19, 2.53) | | **0.0045** |
